# Excessive Inorganic Phosphate Burden Perturbed Intracellular Signaling: Quantitative Proteomics and Phosphoproteomics Analyses

**DOI:** 10.3389/fnut.2021.765391

**Published:** 2022-01-14

**Authors:** Rebecca Hetz, Erik Beeler, Alexis Janoczkin, Spencer Kiers, Ling Li, Belinda B. Willard, Mohammed S. Razzaque, Ping He

**Affiliations:** ^1^Department of Biochemistry, Lake Erie College of Osteopathic Medicine, Erie, PA, United States; ^2^Proteomics and Metabolomics Core, Cleveland Clinic Lerner Research Institute, Cleveland, OH, United States; ^3^Department of Pathology, Lake Erie College of Osteopathic Medicine, Erie, PA, United States

**Keywords:** inorganic phosphate, cytotoxicity, cell signaling, proteomics, phosphoproteomics, alternative splice

## Abstract

Inorganic phosphate (Pi) is an essential nutrient for the human body which exerts adverse health effects in excess and deficit. High Pi-mediated cytotoxicity has been shown to induce systemic organ damage, though the underlying molecular mechanisms are poorly understood. In this study, we employed proteomics and phosphoproteomics to analyze Pi-mediated changes in protein abundance and phosphorylation. Bioinformatic analyses and literature review revealed that the altered proteins and phosphorylation were enriched in signaling pathways and diverse biological processes. Western blot analysis confirms the extensive change in protein level and phosphorylation in key effectors that modulate pre-mRNA alternative splicing. Global proteome and phospho-profiling provide a bird-eye view of excessive Pi-rewired cell signaling networks, which deepens our understanding of the molecular mechanisms of phosphate toxicity.

## Introduction

Inorganic phosphate (Pi) is an essential mineral for life due to its fundamental role in diverse cellular processes. These include nucleic acid synthesis, energy storage, and transfer, cell signal transmission, bone formation, bone growth, and skeletal mineralization (1, 2). Humans routinely intake phosphate through food, which maintains normal musculoskeletal functions. Excessive intake of dietary phosphate, especially from processed food, may result in various health issues, such as dental diseases (3, 4), cardiovascular disease (5), diabetes (6), infertility (7), kidney disease (8), and tumors (9). These disorders are mechanistically mediated by high phosphate-induced pathological calcification (10), oxidative stress (11), cell death, and abnormal signal transduction (12).

Although excessive phosphate (Pi)-dysregulated AKT, mitogen-activated protein kinase (MAPK), and fibroblast growth factor receptor (FGFR) cell signaling pathways have been reported (12–15), the global protein expression and protein phosphorylation profiles perturbed by extracellular Pi remains elusive. Comprehensive analysis of the Pi-related protein phosphorylation landscape can offer a panoramic view of Pi-associated signaling networks, expanding our understanding of how extracellular Pi shapes varied cell behaviors and may provide potential therapeutic targets to manage high phosphate-induced organ damage. Herein, we applied quantitative proteomic and phosphoproteomic strategies to uncover high Pi-mediated alterations in protein expression and protein phosphorylation. The following bioinformatic analyses and literature searching revealed excess Pi-rewired cell signaling networks with extensive cross-talk. Western blot (WB) analysis confirmed a profound change in the regulators that govern the pre-mRNA alternative splicing.

## Materials and Methods

### Materials

Most of the reagents were purchased from Sigma-Aldrich (St. Louis, MO, USA). HeLa cell line was an in-kind gift from Dr. Don Newmeyer (La Jolla Institute for Immunology, San Diego, CA, USA). HEK293 cells, Dimethylsulfoxide (DMSO), XTT Cell Proliferation Assay Kit, and Universal Mycoplasma Detection Kit were purchased from ATCC (Manassas, VA, USA). Dulbecco's modified Eagle's medium (DMEM), Dulbecco's phosphate-buffered saline (DPBS), Ultrapure water, EDTA (0.5 M), Halt™ Protease and Phosphatase Inhibitor Cocktail, Acclaim™ PepMap™ 100 C18 HPLC Column, TMT10plex™ Isobaric Label Reagent Set, High-Select™ Fe-NTA Phosphopeptide Enrichment Kit, UltraPure™ SDS Solution (10%), Pierce™ Rapid Gold BCA Protein Assay Kit, NuPAGE™ Bis-Tris 4–12% precast gels, NuPAGE™ MOPS SDS Running Buffer (20×), NuPAGE™ MES SDS Running Buffer (20×), Restore™ PLUS Western Blot Stripping Buffer, PageRuler™ Plus Prestained 10–250kDa Protein Ladder, Instant Non-fat Dry Milk, Trypsin-EDTA (0.25%), Fetal bovine serum (FBS) and L-Glutamine (200 mM), anti-Phospho-epitope SR proteins antibody (Clone 1H4), and anti-SR protein family protein antibody (Clone 16H3) were obtained from Thermo Fisher Scientific (Carlsbad, CA, USA). Nitrocellulose Membrane, Precision Plus Protein™ Dual Color Standards, and Trans-Blot® Turbo™ RTA Midi Nitrocellulose Transfer Kit were purchased from Bio-Rad (Hercules, CA, USA). Antibodies against AKT1, GAPDH, Phospho-C-JUN (S63), Phospho-C-JUN (S73), C-JUN, Cleaved Caspase-3 (Asp175), and SRSF10 were obtained from Cell Signaling Technology (Danvers, MA, USA). SRPK (D-7) and Caspase-2 (F-2) antibodies were purchased from Santa Cruz Biotechnology (Dallas, TX, USA). Anti-SRPK1 and SRPK2 antibodies were purchased from BD Biosciences (San Jose, CA, USA). SRSF1, SRSF9, and SRSF11 antibodies were purchased from MyBioSource (San Diego, CA, USA). Direct-Blot™ HRP anti-β-actin, HRP Goat anti-mouse IgG, HRP donkey anti-rabbit IgG, HRP mouse anti-rat IgG antibodies, Western-Ready™ ECL Substrate Kit, and antibodies against MYC, Bcl-XS/L, and DYKDDDDK (Flag) Tag were purchased from BioLegend (San Diego, CA). PP1 Catalytic Subunit (PP1C) and Caspase-9 antibodies were purchased from R&D Systems (Minneapolis, MN, USA). The pIRES-EGFP plasmid was obtained from Addgene (Watertown, MA, USA). ORF cDNAs of Wild type (WT) human TRA2A, TRA2B, SRSF1, SRSF2, SRSF3, SRSF4, SRSF5, SRSF6, SRSF7, SRSF11, and SRSF12 cloned into pcDNA3.1+/C-(K)DYK vectors were purchased from Genscript (Piscataway, NJ, USA). FuGENE® HD Transfection Reagent was purchased from Promega (Madison, WI, USA).

### Cell Culture and Treatment

The protocol was followed as detailed by He et al. in an earlier publication (12). Briefly, HEK293 and HeLa were maintained in DMEM (with 1 mM Pi) supplemented with 10% FBS. All cells were maintained at 37°C and 5% CO_2_. Monosodium phosphate (NaH_2_PO4, 2M, pH 6.8) was used as the source of Pi. Cells were treated with an increased amount of Pi for 24 h. For PFA treatment, the cells were pre-treated with PFA at indicated concentrations for 0.5 h followed by Pi treatment at different concentrations in the presence of PFA for 24 h.

### Total Protein Extraction

The protocol was followed as detailed by He et al. in an earlier publication (12). Briefly, the cells were lysed in lysis buffer (50 mM Tris-HCl, pH 7.4, 150 mM NaCl, 0.25% Sodium deoxycholate, 1% NP-40, 0.1% SDS) supplemented with 1× Protease and Phosphatase Inhibitor Cocktail.

### Electrophoresis and Western Blot (WB)

The protocol was followed as detailed by He et al. in an earlier publication (12). Protein concentration was measured by Rapid Gold BCA Protein Assay Kit according to the manufacturer's protocol. A total of 30 μg extracted protein was resolved by electrophoresis and transferred to the nitrocellulose membrane. After blocking with 5% non-fat milk, the membrane was probed with diluted primary antibodies used at the manufacturer's recommended concentrations. Proteins were visualized using HRP conjugated secondary antibodies and chemiluminescence detection. The images were captured by LI-COR C-Digit imaging system (Lincoln, NE, USA).

### Transient Transfection

Cells were transfected with plasmids using FuGENE® HD Transfection Reagent according to the manufacturer's protocol. Total proteins were extracted 24 h posterior to transfection.

### XTT Assay

The protocol was followed as detailed by He et al. in an earlier publication (12). HEK293 cells were transfected with plasmids for 24 h followed by 40 mM Pi treatment. The XTT assay was performed 24 h after high Pi treatment. The average of specific absorbance from biological duplicates was normalized to the 1 mM Pi-treated group. The fold change of relative absorbances was plotted as the mean absorbance ± SEM using GraphPad Prism version 8 software (San Diego, CA, USA).

### Quantitative Proteomics and Phosphoproteomics

HEK293 were treated with physiological (1 mM), pro-survival (10 mM), and pro-death (40 mM) concentrations of Pi for 24 h. Then, the cells were scraped in PBS and the cell pellet was lysed in 8 M urea in 20 mM HEPES buffer pH 8 with phosphatase inhibitors HALT freshly added. The protein concentration of the cell lysates was measured by a BCA assay. Each sample (1 mg of total protein) was reduced with dithiothreitol and alkylated with iodoacetamide before digesting overnight using 200 μg sequencing grade trypsin. Digested peptide samples were desalted using a C18 solid-phase extraction (SPE) column and dried in a Speedvac centrifugal vacuum concentrator (Thermo Fisher Scientific, Waltham, MA, USA). Each sample was dissolved in high-performance liquid chromatography (HPLC) grade water and a quantitative peptide assay was used to measure the peptide concentration. For global proteomics using TMT labels, 25 μg of peptides were taken from each sample and labeled with TMT10plex tags according to the manufacturer's protocol. The TMT labeled samples were desalted and separated using a high pH reversed-phase HPLC method, and the collections were combined into 4 fractions. For phosphoproteomics using TMT, 1 mg of peptides were PO3-enriched using a Hi-select Fe-NTA phospho-enrichment kit. The eluted peptide samples were evaporated in a SpeedVac and desalted using SPE spin columns. The desalted PO3-enriched peptide samples were labeled with TMT labels. The labeled samples were combined without fractionation.

The TMT-labeled samples were analyzed on a ThermoFisher Scientific UltiMate 3000 UHPLC system (ThermoFisher Scientific, Bremen, Germany) interfaced with a ThermoFisher Scientific Orbitrap Fusion Lumos Tribrid mass spectrometer (Thermo Scientific, Bremen, Germany). Liquid chromatography (LC) was performed prior to mass spectrometry (MS)/MS analysis for peptide separation. The HPLC column used is a Thermo Scientific™ Acclaim™ PepMap™ 100 C18 reversed-phase capillary chromatography column (Thermo Fisher Scientific, Waltham, MA, USA) 75 μm x 15 cm, 2 μm, 100 Å. Then, 5 μl volumes of the peptide extract were injected and peptides eluted from the column by a 90-min acetonitrile/0.1% formic acid gradient at a flow rate of 0.30 μl/min and introduced to the source of the mass spectrometer online. Nano electrospray ion source was operated at 2.3 kV. The digest was analyzed using the data-dependent multitask capability of the instrument acquiring full scan mass spectra using a Fourier Transform (FT) Orbitrap analyzer to determine peptide molecular weights and higher-energy collisional dissociation (HCD) MS/MS product ion spectra with the Orbitrap FT analyzer (Thermo Fisher Scientific, San Jose, CA, USA) at 38% normalized collision energy (NCE) to determine both the amino acid sequence and the quantities of the isobaric tags. The MS method used in this study was a data-dependent acquisition (DDA) with a 3 s duty cycle. It includes one full scan at a resolution of 120,000 followed by as many MS/MS scans as possible on the most abundant ions in that full scan. The MS/MS HCD scan starts at 110 m/z with a resolution of 30,000. Dynamic exclusion was enabled with a repeat count of 1 and ions within 10 ppm of the fragmented mass were excluded for a duration of 60 s.

The data were analyzed using Proteome Discoverer V2.3 (Thermo Fisher Scientific, Waltham, MA, USA) with the search engine Sequest-HT which is integrated with the Proteome Discoverer software (Thermo Fisher Scientific, Waltham, MA, USA). The protein sequence database used to search the MS/MS spectra was the Uniprot mouse protein database containing 25,035 entries with an automatically generated decoy database (reversed sequences). The protease was set to full activity trypsin with a maximum of two missed cleavages. Oxidation of Methionine and acetylation of protein N-terminus were set as dynamic modifications and carbamidomethylation of cysteine, TMT6plex of Lysine, and peptide N-terminus were set as static modifications. The precursor mass tolerance for these searches was set to 10 ppm and the fragment ion mass tolerance was set to 0.02 Da. Keratins were known contaminants and were excluded from identified proteins. A false discovery rate (FDR) was set to 1% for both peptide and protein identification and calculated using the number of identified peptides/proteins from the decoy database divided by the total number of identified peptides/proteins. Two peptides were required for positive protein identification to decrease the chance of false discovery by a random match.

Relative quantitation of the samples labeled by different isobaric tags was done by the Reporter Ions Quantifier node in Proteome Discoverer using the intensity of the reporter ions from MS/MS scans. The m/z tolerance of the reporter ions was set to 20 ppm, and the ion selection was set to the most confident centroid. Quantitative values were normalized by the total amount of peptide in each label channel. The peptide used for quantification was set to Unique + Razor, and the precursor Co-isolation threshold was set to 50%. Razor-peptides are non-unique peptides and these are assigned to the protein group containing the largest number of other peptides, according to Occam's razor principle.

### Bioinformatics

The high-qualified (*P* < 0.05 from three biological replicates) differentially expressed proteins with at least two matching peptides and phosphopeptides with >2-fold change between 1 and 10mM Pi-treated cells, and 1 and 40mM Pi-treated cells, were the input dataset for the bioinformatic analyses.

#### Kyoto Encyclopedia of Genes and Genomes (KEGG) Mapping

The Kyoto Encyclopedia of Genes and Genomes (KEGG) pathway map is a manually drawn graphical diagram showing metabolic, signaling, and other molecular interaction/reaction networks (16). The differential hits were uploaded to KEGG Mapper (https://www.genome.jp/kegg/tool/map_pathway1.html) to map specific proteins in KEGG signaling pathways.

#### HuRI Mapping

HuRI is a reference map of the human binary protein interactome, documenting human “all-by-all” binary protein interactions based on experimental validation (high-throughput yeast two-hybrids) and literature curation (17). The differential hits were uploaded to the Human Reference Interactom (HuRI) database (http://www.interactome-atlas.org/search) to acquire the Pi-associated protein interactome.

#### Ingenuity Pathway Analysis (IPA)

Ingenuity pathway analysis is a web-based software application that enables analysis, integration, and understanding of data from gene expression, miRNA, and SNP microarrays, as well as metabolomics, proteomics, and RNAseq experiments (18–21). The proteins and protein phosphorylation significantly dysregulated by high Pi treatment identified in the proteome and phosphoproteome were investigated using IPA software [outsource service provided by QIAGEN (Germantown, MD, USA), https://www.qiagen.com/ingenuity]. This analysis used a pre-made significance test as a cut-off (*p* < 0.05) for all fold change values, to note enrichment in IPA. The analysis examines genes in the dataset known to affect each biological function and compares their direction of change to what is expected from the literature. Phospho-proteomic core analysis is performed to identify significantly associated canonical pathways, predicted upstream regulators, the top predicted diseases and bioFunctions associated with differentially expressed gene set and molecular networks.

### Statistics

An unpaired student's *t*-test was used to compare means between control and high Pi-treated groups. A value of *P* < 0.05 was considered statistically significant. ^*^P < 0.05, ^**^P < 0.01, ^***^P < 0.001, ^****^P < 0.0001. GraphPad Prism version 8 software was used to perform the statistics (San Diego, CA, USA).

## Results

### Proteomic and Phosphoproteomic Profiling of High Pi-treated Cells

**Figure 1:**
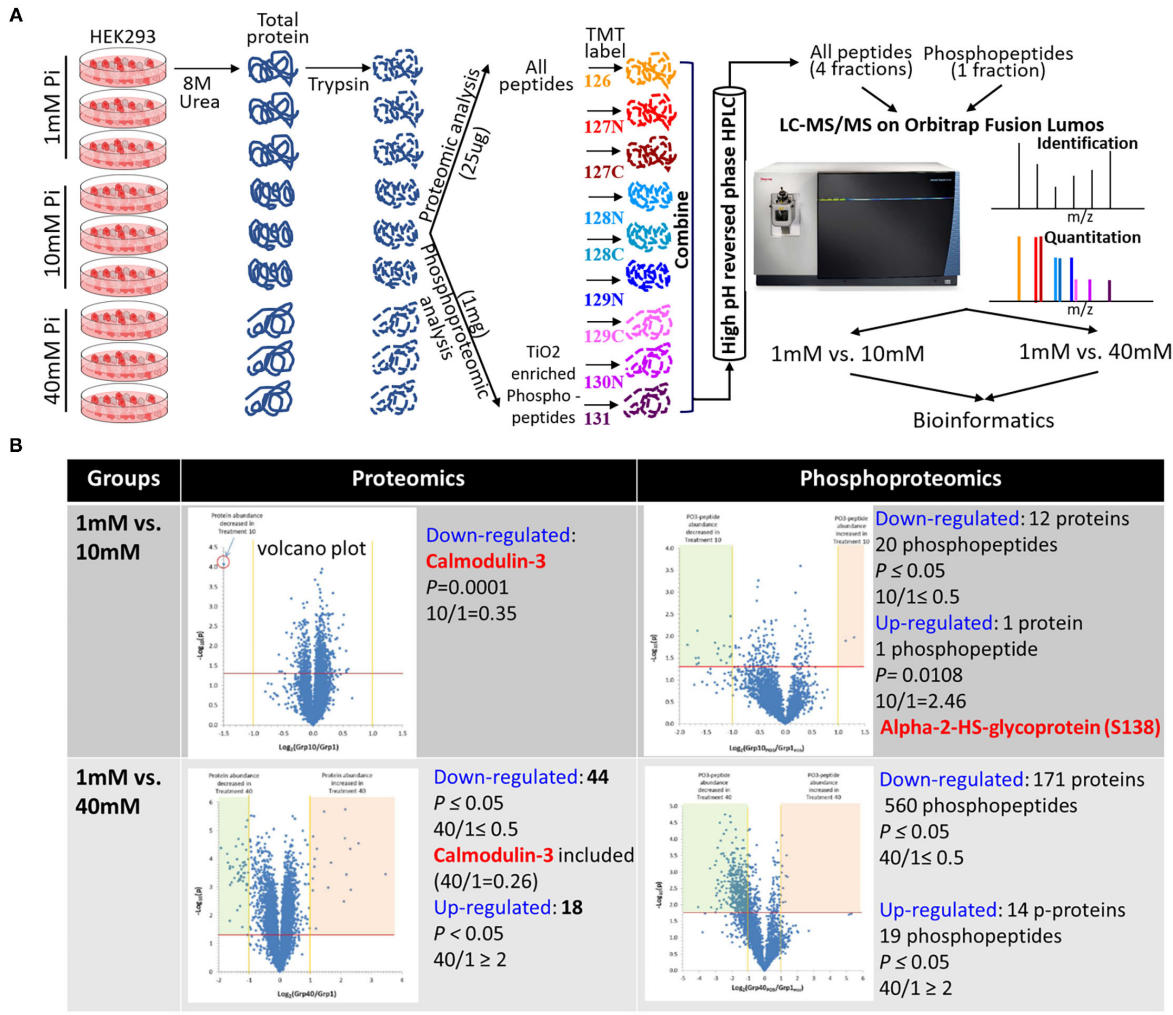
Comparison of protein abundance and protein phosphorylation between non-treated and high Pi-treated cells by quantitative proteomics and phosphoproteomics. **(A)** Experimental flow chart. **(B)** Summary of differential hits between groups. The embedded volcano plots demonstrate statistical significance (P-value, y-axis) vs. magnitude of change (fold change, x-axis).

To understand the complex networks and functions coordinated by protein phosphorylation, we adopted proteomic and phosphoproteomic platforms to analyze Pi-related protein and protein phosphorylation change globally and quantitatively in HEK293 cells (Figure 1A).

The cells were treated with 1 mM (physiological), 10 mM (pro-survival), and 40 mM (pro-death) Pi for 24 h. We applied an abnormally high concentration of Pi in the acute phosphate toxicity cell model to determine the immediate cytotoxic effects, as detailed in our earlier publication (12). The extracted protein was digested and labeled prior to LC-MS/MS analysis. Each treatment has 3 biological replicates. A total of 4,704 proteins were identified with at least two matching peptides (Supplementary Table 1), and the quantitation result is given in Supplementary Table 2. A total of 5,041 phosphopeptides were identified, within which 4,249 peptides had quantitative values (Supplementary Table 3). We thereafter compared the global protein and protein phosphorylation changes between 1 and 10 mM, and 1 and 40 mM Pi-treated groups and set ≥2-fold change and *P* ≤ 0.05 (*n* = 3) as cut-off values. Compared to 1mM Pi-treated groups, 10 mM Pi treatment decreased the level of one protein (Calmodulin-3, highlighted in Supplementary Table 4) and 20 phosphopeptides from 12 proteins (highlighted in red in Supplementary Table 5), increased the abundance of one phospho-peptide (S138) from Alpha-2-HS-glycoprotein (highlighted in yellow in Supplementary Table 5). In contrast to 10, 40 mM Pi caused more pronounced global protein expression and protein phosphorylation changes. The treatment resulted in the downregulation of 44 proteins (highlighted in red in Supplementary Table 6) and upregulation of 18 proteins (highlighted in yellow in Supplementary Table 6). It led to 560 underrepresented phosphopeptides from 171 proteins (highlighted in red in Supplementary Table 7) and 19 overrepresented phosphopeptides from 14 proteins (highlighted in yellow in Supplementary Table 7). The differential expressed proteins and phosphopeptides are summarized in Figure 1B.

### Bioinformatic Analysis of High Pi-perturbed Cell Signaling Networks

The differentially expressed proteins and protein phosphorylation with high quality (as described at Bioinformatics) are the input dataset for the following bioinformatic analyses.

#### KEGG Mapping

The analysis mapped differential protein and phosphorylation hits between 1 and 40 mM Pi-treated cells to enriched pathways, including spliceosome, complement, and coagulation cascades, Rap1 signaling pathway for proteome, and spliceosome, RNA transport, and pathways in cancer for phosphoproteome. High Pi impairment of spliceosome assembly is exemplified in Supplementary Figure 1A.

#### HuRI Mapping

The analysis identified protein-protein interaction networks of 40mM Pi-mediated reduction of protein expression and protein phosphorylation. The molecular interaction networks are involved in RNA splicing by proteomics and phosphoproteomics (Supplementary Figure 1B).

#### IPA

##### Canonical Pathways

**Figure 2:**
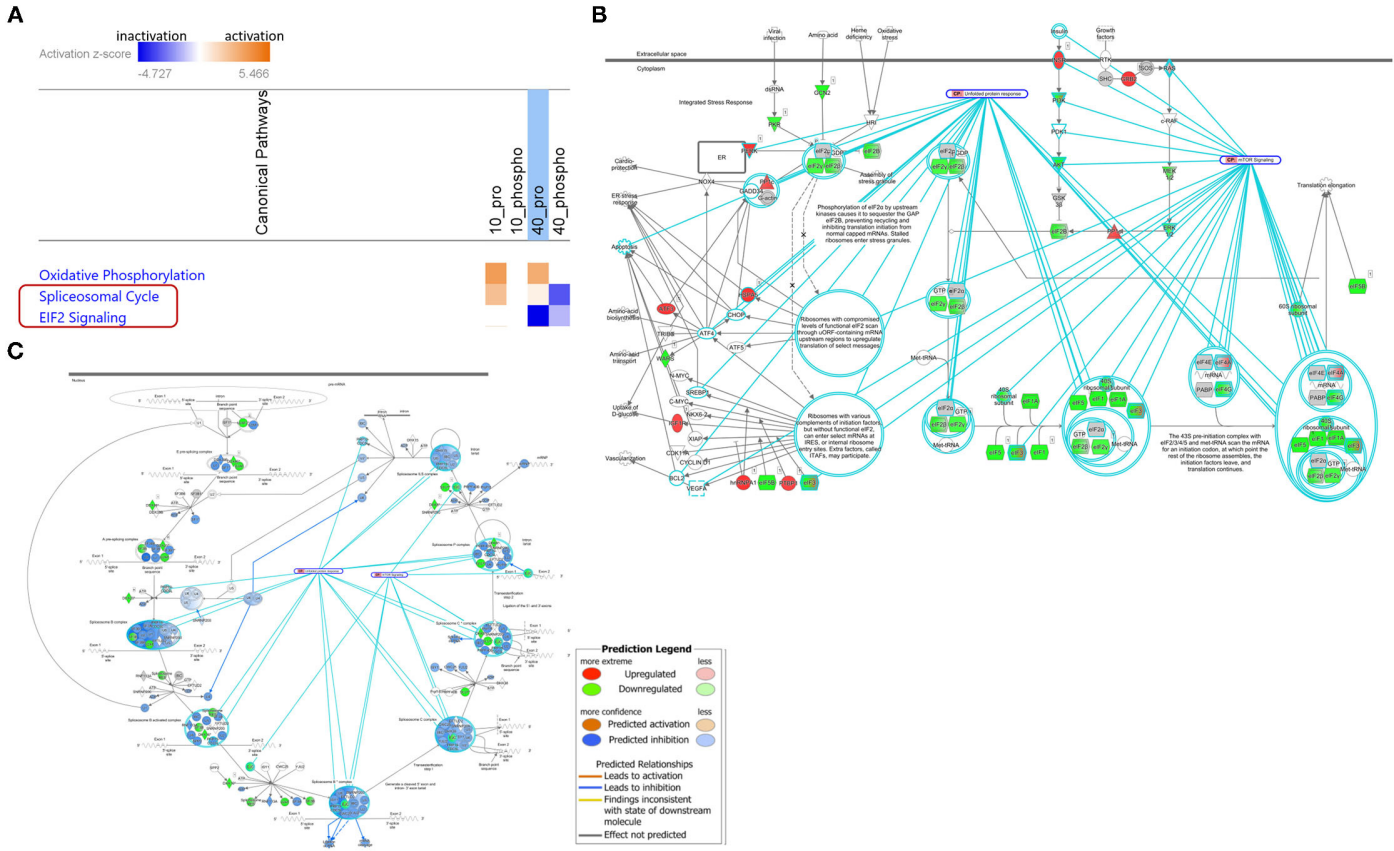
High Pi-dysregulated significant pathways by IPA Canonical Pathways analysis. **(A)** Heatmap of the comparison analysis. Orange: pathways with positive z-scores; Blue: pathways with negative z-scores; White: pathways that have a z-score of 0, indicating that the differential gene expression data did not allow for a clear determination of the activity prediction. 10_Pro: 10 mM Pi treatment by proteomic analysis; 10_Phospho: 10 mM Pi treatment by phosphoproteomic analysis; 40_Pro: 40 mM Pi treatment by proteomic analysis; 40_Phospho: 40mM Pi treatment by phosphoproteomic analysis. EIF2 signaling **(B)** and spliceosomal cycle **(C)** pathways were colored using the Molecular activity predictor. Molecules in red and green indicate high Pi upregulated and downregulated protein expression or phosphorylation, respectively.

The core analysis identified the IPA canonical pathways that were significantly enriched from the dataset. Fisher exact test was used to determine significant p-values and the association of the dataset with canonical pathways. In addition, pathways were predicted to be activated or inhibited based on the entire dataset provided to IPA. The analysis identified significantly dysregulated pathways in 40mM Pi-treated cells. A Heatmap of the comparison analysis showed significant inactivation of EIF2 signaling by proteomic analysis and spliceosomal cycle by phosphoproteomic analysis (Figure 2A). Specific pathways, such as EIF2 signaling (Figure 2B) and spliceosomal cycle (Figure 2C), were colored using the Molecular activity predictor in IPA.

##### Upstream Regulators

Ingenuity pathway analysis (IPA) core analysis allows for predicting upstream regulators that may be responsible for the gene expression changes observed in the dataset based on the information from the IPA knowledge base. Network maps for specific upstream regulators were colored by MAP and overlaid with specific canonical pathways. Supplementary Figure 2A displays high Pi downregulated key regulators of RNA splicing (SRPK1/2, CLK1) and cell signaling (CSNK2A1, CDK1/6). The affected regulating networks are depicted in Supplementary Figures 2A–H.

##### Disease and Function Analysis

The Diseases and Functions Analysis identifies downstream effects that are expected to increase or decrease, given the observed gene expression changes in the dataset. It is based on the expected causal effects, derived from the literature compiled in the Ingenuity Knowledge Base, between genes and functions. The analysis examines genes in the dataset that are known to affect functions and uses the expected causal effects of the genes derived from the literature to issue a prediction for each function, based on the direction of change in gene expression. The z-score captures the direction of change. In line with our previous findings (12), proteomics and phosphoproteomics revealed that 10mM Pi treatment increased cell viability and decreased cell death/apoptosis, while 40mM Pi treatment enhanced cell death and mitigated cell survival (Supplementary Figure 3).

##### Network Analysis

Network view displays an interactive graphical representation of the interrelationships between molecules. The analysis demonstrated complicated molecular networks regulating splicing and processing of RNA in 40mM Pi-treated cells by phosphoproteomics (Supplementary Figure 4).

### Literature-Based High Pi-impacted Cellular Processes

**Figure 3:**
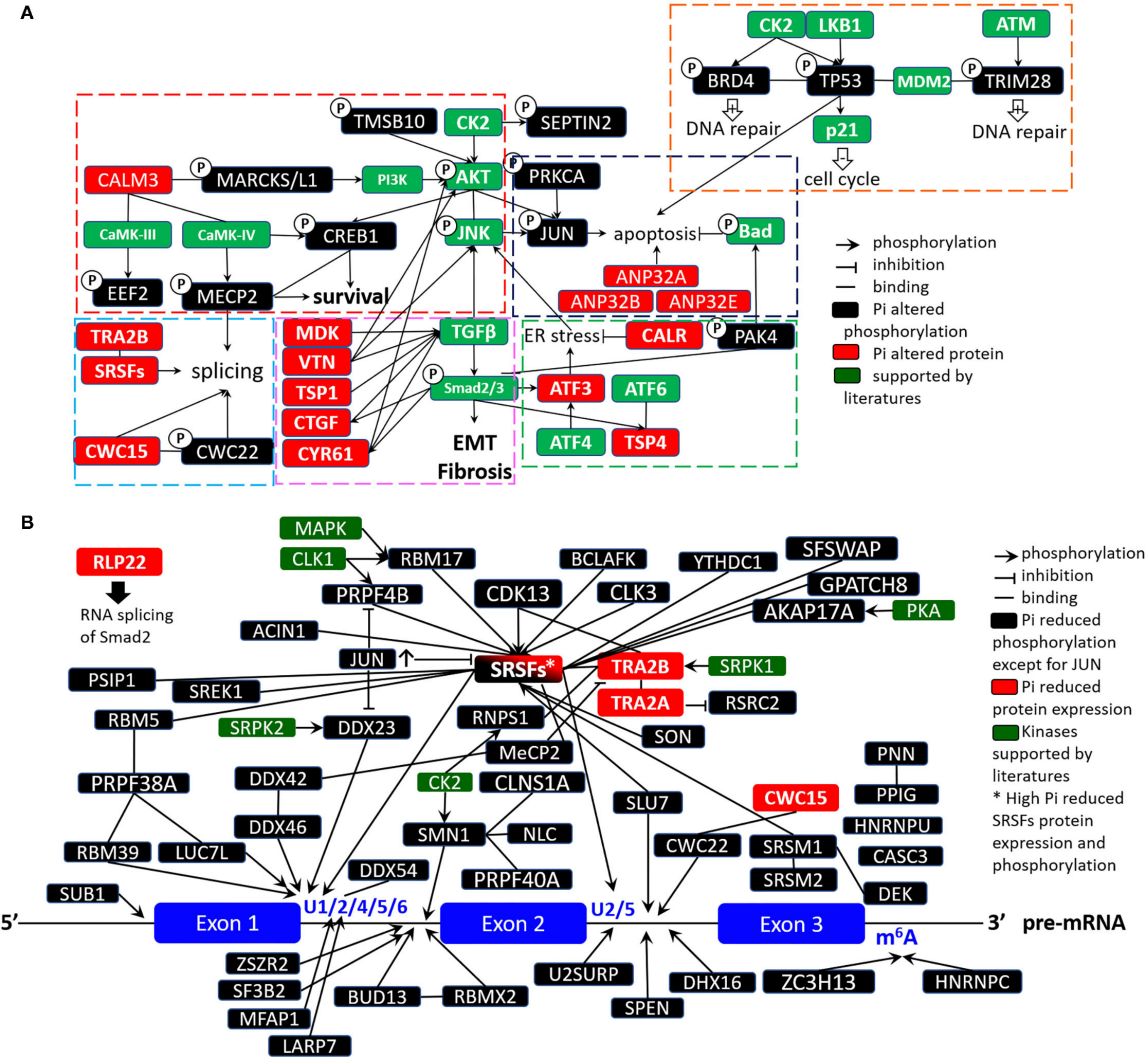
High Pi-perturbed signaling networks based on literature. **(A)** Molecular cross-talks among various pathways. **(B)** High Pi-dysregulated mRNA splicing.

In parallel, the differential hits resulting from different concentrations of Pi treatment by proteomics and phosphoproteomics were searched against published data. As shown in Supplementary Figure 5, the literature search revealed that the dysregulation of protein expression and phosphorylation by high Pi suppresses Calcium signaling, CREB signaling, cell cycle progression, and pre-mRNA alternative splicing. It promotes TGF-β signaling, ER stress, and apoptosis. Cross-talk among the Pi-rewired signaling pathways is demonstrated in Figure 3A. Notably, mRNA alternative splicing is the most extensively affected by high Pi treatment (Figure 3B).

### High Pi-mediated the Dysregulation of MRNA Splicing

**Figure 4:**
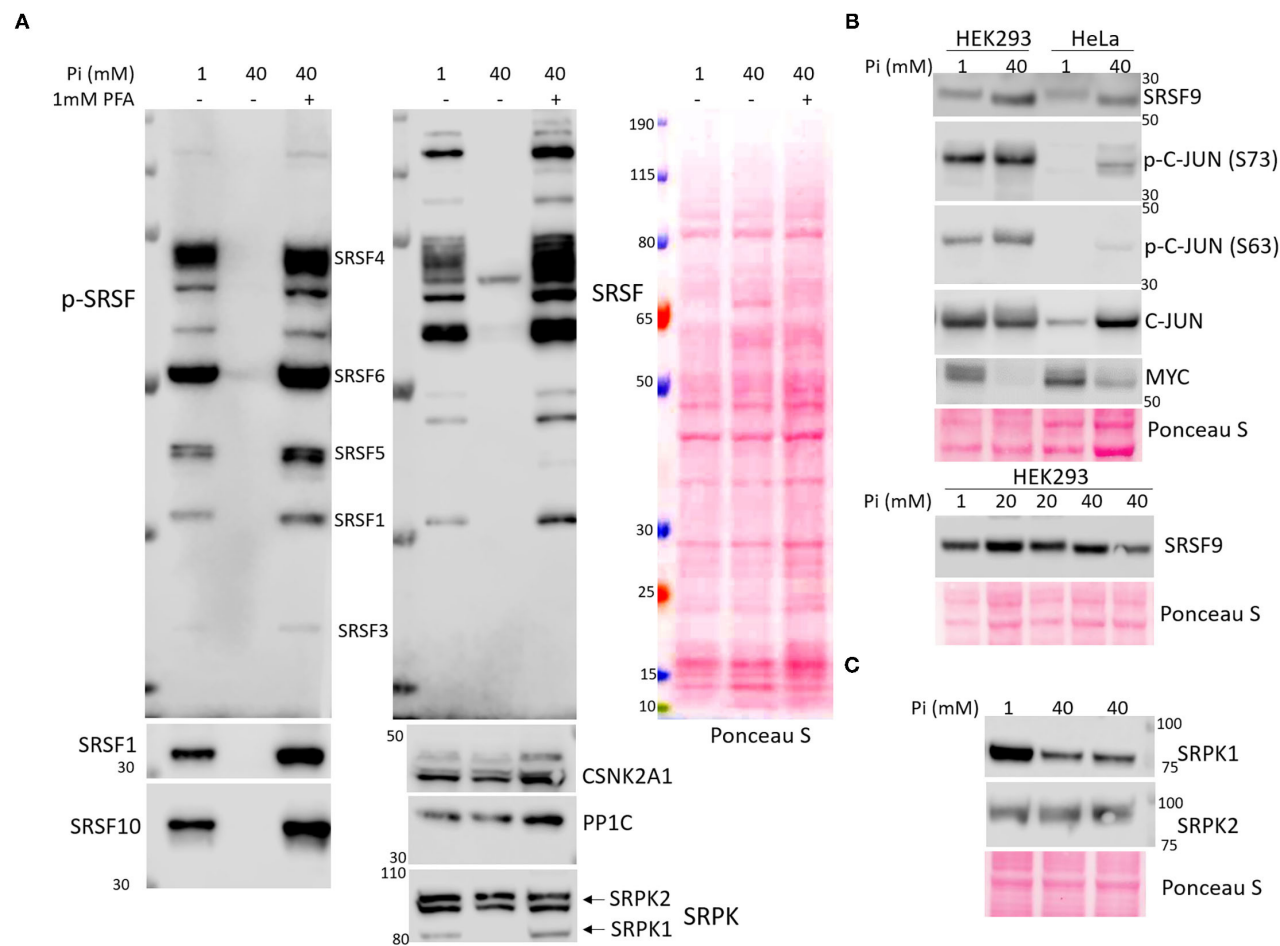
Elevated extracellular phosphate-mediated dysregulation of the effectors in mRNA alternative splicing process by Western blot (WB) analysis. HEK293 and HeLa cells were grown to 80–90% confluence followed by treatment with increased NaH_2_PO_4_ (Pi) for 24 h. In the PFA treated group **(A)**, HEK293 cells were pre-treated with 1mM PFA for 0.5 h followed by 40mM Pi treatment in the presence of PFA for 24 h. Total protein (30 μg) extracted from each treatment was used for WB analysis with the primary anti-phospho SRSFs, SRSFs, CSNK2A1, PP1C, SRPK (recognizing both SRPK1 and SRPK2) antibodies in **(A)**, anti-phospho c-JUN (S63), phospho c-JUN (S73), c-JUN, SRSF9, and MYC antibodies in **(B)**, and anti-SRPK1and SRPK2 antibodies in **(C)**. The Ponceau S stain of the membrane was used as a loading control.

Bioinformatics (Figure 2, Supplementary Figures 1, 5) and literature searching (Figure 3) congruently show the high Pi-elicited differentially expressed and phosphorylated proteins enriched in pathways that regulate pre-mRNA splicing and spliceosome assembly. Among the 40mM Pi downregulated proteins and protein phosphorylation, 12 out of 18 (66.7%) proteins and 63 out of 187 (33.7%) phosphorylated proteins participate in RNA splicing. To validate the proteomic and phosphoproteomic data, we examined the expression and phosphorylation of RNA splicing effectors, especially SR proteins (SRSFs) and SR protein kinases (SRPK), and their master regulators, CSNK2A1 and JUN (as indicated in Supplementary Figures 2, 4, 5), PP1C (22) and MYC (23) (as noted in literature). WB analysis verified universal decline of SRSFs' expression (SRSF1, SRSF4, and SRSF10) and phosphorylation (SRSF1, SRSF3, SRSF4, SRSF5, and SRSF6) induced by excessive extracellular Pi (Figure 4A). We observed a slight molecular weight (Mw) shift (possibly due to high Pi-mediated dephosphorylation) of SRSF9 in 40mM Pi-treated cells (Figure 4B). We also observed reduced SRPK1, but not SRPK2, in high Pi-treated cells (Figures 4A,C). The high Pi-suppressed SRSFs and SRPK1 were abolished by the inhibition of Pi intake with phosphonoformic acid (PFA), an inhibitor of sodium/phosphate (Na/Pi) co-transporters (24). Regarding the upstream regulators of SRSFs and SRPKs, we validated increased phosphorylation of c-JUN at Serine (S) 63 and S73 and detected decreased expression of MYC induced by high Pi (Figure 4B). However, we found no expression changes in CSNK2A1 and PP1C in the cultured cells exposed to elevated Pi (Figure 4A).

### High Pi-induced Cell Death Is Independent of Aberrant MRNA Splicing

**Figure 5:**
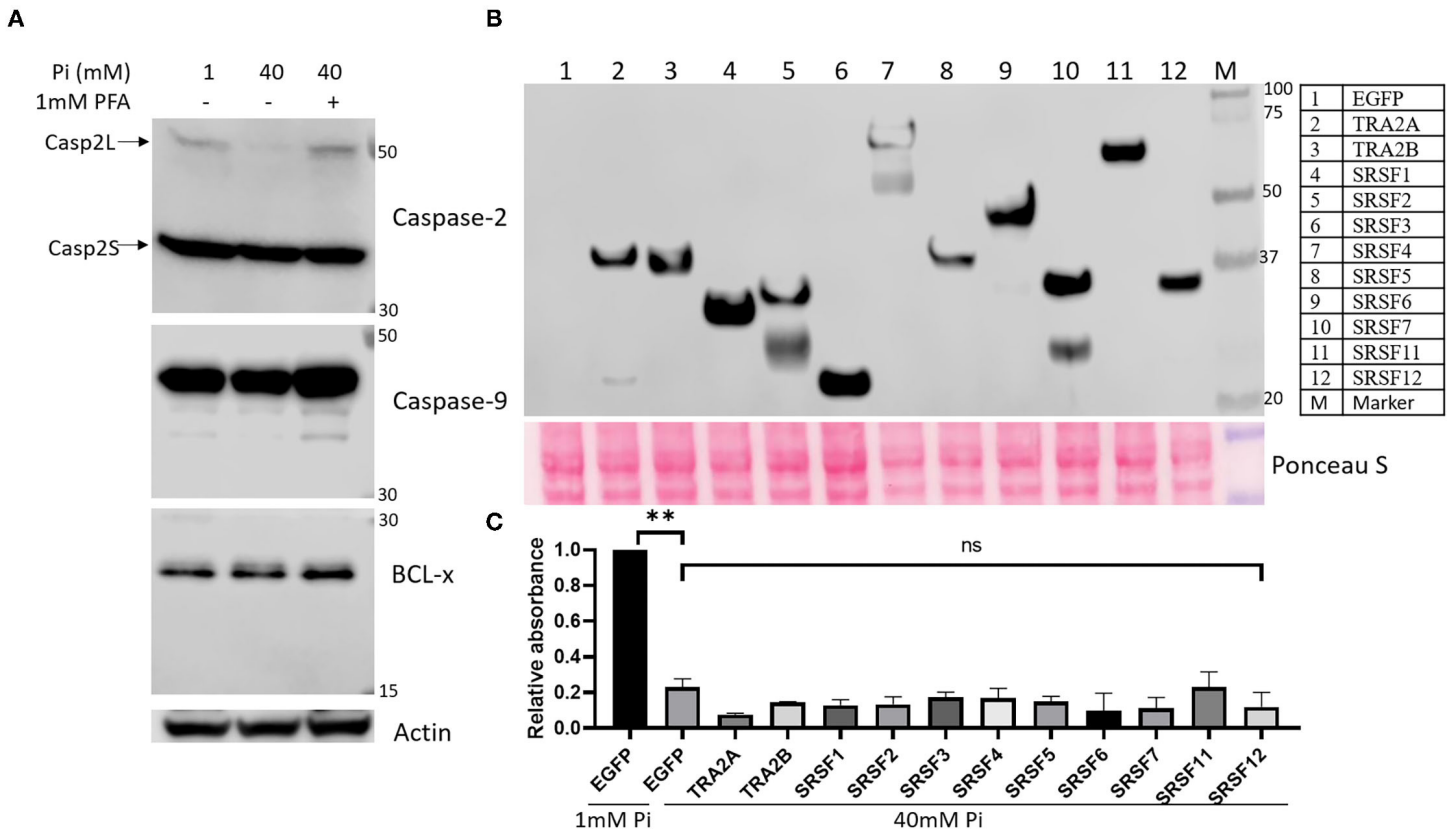
**(A)** High Pi treatment reduces alternative splicing of Caspase-2 but has no effect on Caspase-9 and BCL-x splicing in HEK293 cells. Actin was used as a loading control. **(B)** Over-expression of SR proteins. HEK293 cells were transfected with pIRES2-EGFP (negative control) and pcDNA3.1-Flag vectors harboring WT full-length cDNA sequence of human SRSF1-7, SRSF11, SRF12, TRA2A, and TRA2B. Cells were harvested 24 h after transfection, and 15μg total protein was analyzed by WB with anti-Flag primary antibody. PageRuler™ Plus Prestained 10-250 kDa Protein Ladder was loaded as protein molecular weight marker. Ponceau S stain of the membrane was used as a loading control. **(C)** WT SR protein complementation does not prevent high Pi-induced cell death by XTT analysis. After being transfected with plasmids for 24 h, HEK293 cells were treated with 1 mM and 40 mM Pi for 24 h followed by XTT assay. The data were represented as means ± SEM from two independent experiments. An unpaired Student t-test was used to compare means between 1 and 40 mM Pi-treated cells transfected with the pIRES2-EGFP plasmid, and 40 mM Pi-treated cells transfected with pIRES2-EGFP plasmid vs. vectors encoding SR proteins. **P < 0.01; ns, not significant.

Western blot (WB) analysis displayed 40mM Pi suppressed splicing of Caspase-2 by showing decreased Caspase-2 long isoform (Casp2L) compared to Caspase-2 short isoform (Casp2S), but no splicing alterations in Caspase-9 nor Bcl-x (Figure 5A).

As global proteomic and phosphoproteomic profiling, in line with WB verification, demonstrated a high Pi-mediated decrease in the expression and phosphorylation of key mRNA splicing regulators, we wanted to know if the complementation of those regulators could rescue the cells cultured in a high Pi medium. HEK293 cells were transfected with plasmids encoding wild-type (WT) SR proteins (SRSF1-7, SRSF11, SRF12, TRA2A, and TRA2B) fused with Flag tag. WB analysis confirmed the successful over-expression of each regulator at correct Mws by using an antibody against the Flag tag (Figure 5B). After 24 h following transfection, cells were treated with 40mM Pi for another 24 h followed by XTT analysis of cell survival. XTT analysis showed that high Pi-induced cell death could not be significantly prevented by over-expressing SR proteins (Figure 5C), suggesting minor roles of defective mRNA splicing in excess Pi-mediated cell damage.

## Discussion

### Use of Phosphoproteomics to Study High Pi-rewired Signaling Networks

We previously showed high phosphate-mediated damage in mice kidneys (25), and in cells that originated from kidneys, such as HEK293 (12). The *in vitro* study showed that extracellular increased Pi elicited a profound change in protein phosphorylation, which is one type of post-translational modification (PTM). It is the core driving force for cell signaling and orchestrates several cellular processes (26). Intracellular signaling usually involves reversible protein phosphorylation regulated by the activity of kinase that uses ATP as a substrate to phosphorylate signaling molecules and the activity of phosphatase that catalyzes the transfer of the Pi from a phosphoprotein to a water molecule (27). Pi may serve as an important regulator of phosphatase in different organisms (28). Hence, the alteration of extracellular Pi may affect the activity of phosphatase and regulate the overall cell signaling network. Beck's group established the investigation of phosphate-controlled cellular response by quantitative proteomics (2, 29, 30). They used cleavable isotope-coded affinity tag reagents to identify and quantitate protein expression differences in phosphate-treated murine MC3T3-E1 osteoblast cells. They found a nearly two-fold increased abundance of Cyclin D1 when cells treated with 10mM NaH_2_PO4 for 24 h were compared to Na_2_SO4 treated control cells (30). However, these studies focused on Pi-related transcriptomics and proteomics instead of PTMs, such as phosphorylation. Therefore, global phosphorylation dynamics perturbed by extracellular phosphate remain elusive. The phosphoproteomic strategy has been proven to successfully analyze a drug or pathogen-induced overall change in phosphorylation events occurring in a cell at different time phases (31–37). A phosphoproteomics-based investigation of the global phosphorylation and protein abundance landscape of phosphate toxicity can help us understand the connections between the known Pi-related cell signaling pathways and expand our view of phosphate-associated signaling networks that induce direct cytotoxicity. Thus, we adopted quantitative proteomic and phosphoproteomic platforms to analyze Pi-mediated protein and protein phosphorylation change (Figure 1A). The analysis revealed significant changes in both global protein expression and protein phosphorylation induced by extremely high concentrations of extracellular Pi, especially for 40 mM Pi-treated cells (Figure 1B).

### Bioinformatics and Literature Searching Reveals the Cross-Talk Among High Pi-perturbed Cell Signaling Pathways

To acquire an extensive and in-depth view of high Pi-dysregulated cellular processes, we harnessed different bioinformatic tools to study Pi-altered pathways (by KEGG mapping and IPA), protein-protein interaction network (by HuRI mapping), and master regulators (by IPA). These bioinformatic analyses revealed elevated Pi-rewired Rap1 signaling and EIF2 signaling pathways (Figure 2A), as well as high Pi-mediated dysregulation of pre-mRNA alternative splicing and the spliceosomal cycle (Supplementary Figure 1, Figure 2). Further literature search confirmed not only excess Pi-perturbed mRNA splicing, but also displayed high Pi-dysregulated calcium signaling, CREB signaling, cell cycle progression, TGF-β signaling, ER stress, and apoptosis. The latter three biological processes induced by high Pi were also validated in our earlier study (12). Moreover, Pi-related cellular processes and pathways synergistically contribute to high Pi's cytotoxicity by extensive and intricate cross-talk (Figure 3A).

### Pi Cytotoxicity and Defective mRNA Splicing

We focused on high Pi-mediated aberrant mRNA splicing because both bioinformatics and literature review (38) revealed high Pi caused differential protein expression and phosphorylation highly enriched in the pre-mRNA splicing process. To the best of our knowledge, there is no study focusing on phosphate's toxic effects on RNA splicing. The assembly of the spliceosome and the subsequent splicing requires serial phosphorylation and dephosphorylation of essential factors known as SR proteins (38). Impressively, proteomic analysis revealed that the majority of SR protein family members (SRSF1-8,12 and TRA2A/B) showed lower abundance and hypo-phosphorylation in 40mM Pi-treated cells compared to 1mM Pi-treated cells. Kinases are known to phosphorylate the RS (Arginine/Serine)-rich regions of SR proteins and other spliceosomal proteins include members of the Cdc2-like kinase family (CLK1-3, PRPF4B) and SRPK family (SRPK1-3), CDK13, and DNA topoisomerase 1 (39). WB analysis verified the reduction in protein expression and phosphorylation in several SR proteins and demonstrated low expression of one of the key kinases of SR proteins, SRPK1 (Figure 4). Since dephosphorylation by phosphatase (mainly PP1 and PP2A) in SR proteins is also required for splicing activity (40), we tested PP1's expression in high-Pi treated cells. However, WB did not detect a significant change in the PP1 level (Figure 4A). Beyond kinases and phosphatases, splicing effectors can also be transcriptionally modulated. Katiyar et al. reported that c-JUN could directly impair mRNA splicing by downregulating the expression of over 50 genes controlling mRNA processing and splicing, including SRSFs (41). Oncoprotein MYC could transcriptionally upregulate the expression of the SR protein splicing factor, SRSF1, in lung cancer cells, which triggered mRNA splicing of a series of kinases and facilitated oncogenic signaling (23). Indeed, we found increased phosphorylation of JUN and a reduced amount of MYC in elevated Pi-treated cells (Figure 4B). We applied the HeLa cell line, a widely used homogenous experimental cell line in the published research works, in the verification study to ensure the consistency and the reproducibility of WB data. Therefore, we used HEK293 cells as primary and HeLa cells as secondary cell-based model systems in this study. Taken together, our findings suggest increased phosphate exposure gives rise to defective mRNA splicing in cultured cells.

Next, we wanted to determine whether high Pi-associated alteration of mRNA splicing contributes to phosphate cytotoxicity. Excessive Pi-induced apoptosis is one of the fundamental mechanisms of Pi-related tissue damage (11, 12). The alternative pre-mRNA splicing regulated programmed cell death has been well documented (42–44). As summarized by Schwerk and Schulze-Osthoff (43), several pro-/anti-apoptotic effectors are regulated by alternative splicing that generates different protein isoforms with different and sometimes even opposite functions during apoptosis. For instance, SR proteins, SRSF1 and SRSF2, promote skipping of exon 9 in the *Caspase-2* gene, causing an increased expression of pro-apoptotic Capase-2 long isoform (Casp2L) and thus enhancing apoptosis. In contrast, hnRNP facilitates the inclusion of exon 9 in *Caspase-2*, generating a premature stop codon and that leads to the expression of pro-survival Capase-2 short isoform (Casp2S) (45). Consistently, we found elevated Pi-induced depression of SRSF1, SRSF2, and the subsequent decrease of pro-apoptotic Casp2L (Figure 5A), indicating an anti-apoptotic role of high Pi-dysregulated mRNA splicing. Researchers also found that the apoptosis inducer, ceramide, could dephosphorylate SR proteins by activating PP1 phosphatase, which mediated the increase of pro-apoptotic splicing variants of Caspase 9 and Bcl-x(s) (46). However, we did not detect excess Pi-mediated alterations in the alternative splicing of Caspase-9 and Bcl-x (Figure 5A). The over-expression of SR proteins could not prevent high Pi-induced cell death (Figure 5B), suggesting high Pi-mediated suppression of SR proteins plays a minor role in excessive Pi-induced apoptosis. Collectively, this data indicates cell damage caused by excess Pi is not primarily ascribed to the changes in mRNA alternative splicing.

### Limitations

We previously showed the effects of phosphate toxicity on kidney tissues (25) and cells obtained from the kidney (HEK293) (12). Hence, we used HEK293 cells as primary cells to investigate cell signaling networks rewired by elevated phosphate in this study. However, since different cell lines' responses to increased extracellular Pi may vary (13, 15, 47–49), the proteomic and phosphoproteomic landscapes in high environmental Pi from one cell line may not be applicable to other cell lines. Systematic analysis of other lines derived from various tissues cultured in medium with different concentrations of Pi is desired to globally profile Pi-associated proteomic and phosphoproteomic signatures. This study focuses on acute Pi-mediated cytotoxicity by exposing cells with a high concentration of Pi for 24 h. Chronic effects (for instance, 3–7 days treatment) of elevated Pi may generate varying results on proteomics and phosphoproteomics. Again, in our study, we used the non-physiologic concentration of Pi to document the immediate cytotoxic effects of Pi by exposing the cells to the toxic range of Pi. We believe such an approach is likely to provide mechanistic insights into acute cytotoxicity.

We did not observe the rescue effects with the over-expression of plasmid-encoded WT SR proteins in high Pi-treated cells. The transfection of a single target may cause it. Several SR proteins may play combinational roles in regulating mRNA alternative splicing in high Pi-treated HEK293 cells. Therefore, co-transfection of multiple SR proteins in excessive Pi-treated cells may assist the understanding of the relationship between RNA splicing and apoptosis in the context of phosphate toxicity.

## Conclusion

Underlying the broad spectrum of phosphate cytotoxicity is the intricate high phosphate-perturbed cell signaling networks and cellular processes as revealed by quantitative proteomic and phosphoproteomic analyses. The bioinformatics and functional assays determined abnormal pre-mRNA splicing as a novel mechanism of phosphate-induced cytotoxicity. An in-depth study of how aberrant alternative splicing contributes to phosphate toxicity will open another window to fully understand the high phosphate-related pathologies, and likely to provide therapeutic clues to manage phosphate toxicity-associated organ damages.

## Data Availability Statement

The data presented in the study are deposited in the PRIDE repository, accession number PXD026301, and 10.6019/PXD026301.

## Author Contributions

PH and MR: conceptualization. RH and EB: methodology. PH and LL: software. RH and PH: validation. PH: formal analysis, writing—original draft preparation, supervision, project administration, and funding acquisition. AJ, SK, and BW: investigation. LL and BW: resources. EB, AJ, and SK: data curation. RH and MR: writing—review and editing. All authors have read and agreed to the published version of the manuscript.

## Funding

This research was funded by LECOM internal Seed Grants, grant number COM-20-21. The Fusion Lumos instrument was purchased *via* an NIH shared instrument grant, 1S10OD023436-01.

## Conflict of Interest

The authors declare that the research was conducted in the absence of any commercial or financial relationships that could be construed as a potential conflict of interest.

## Publisher's Note

All claims expressed in this article are solely those of the authors and do not necessarily represent those of their affiliated organizations, or those of the publisher, the editors and the reviewers. Any product that may be evaluated in this article, or claim that may be made by its manufacturer, is not guaranteed or endorsed by the publisher.
