# Supplementary material for: Excessive Inorganic Phosphate Burden Perturbed Intracellular Signaling: Quantitative Proteomics and Phosphoproteomics Analyses
Source: Front Nutr. 2022 Jan 14;8:765391. doi: 10.3389/fnut.2021.765391 (PMC8795896; doi:10.3389/fnut.2021.765391)
Supplement: Supplementary file 8 [file Data_Sheet_1.DOCX]

Supplementary Material

## Supplementary Figures


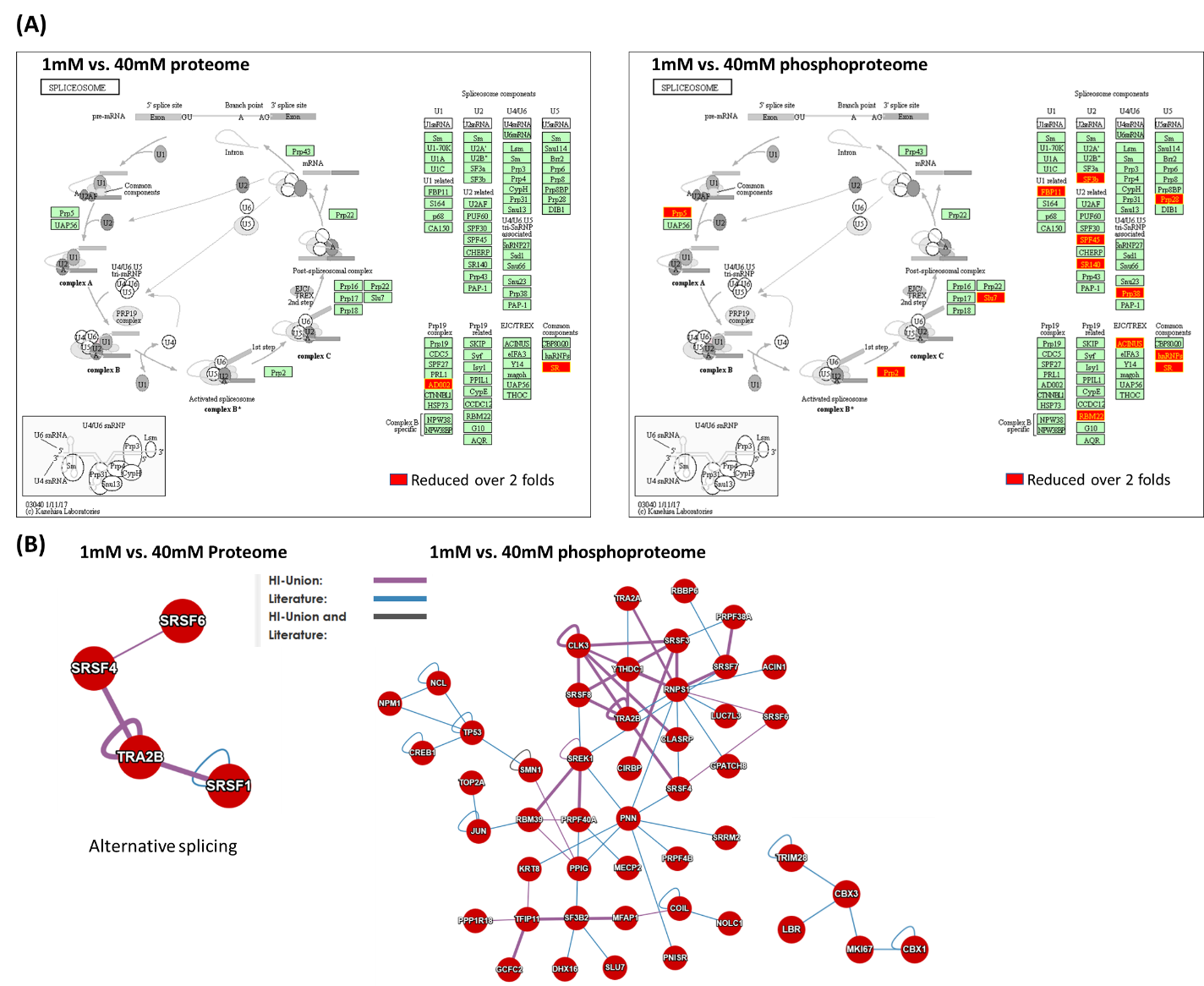


**Supplementary Figure 1.** (**A**) KEGG mapping of high Pi-mediated reduction of proteins (left panel) and protein phosphorylation (right panel) enriched in the assembly of spliceosome. The differential hits are highlighted in red. (**B**) HuRI mapping of high Pi-mediated reduction of proteins (left) and protein phosphorylation (right).

**
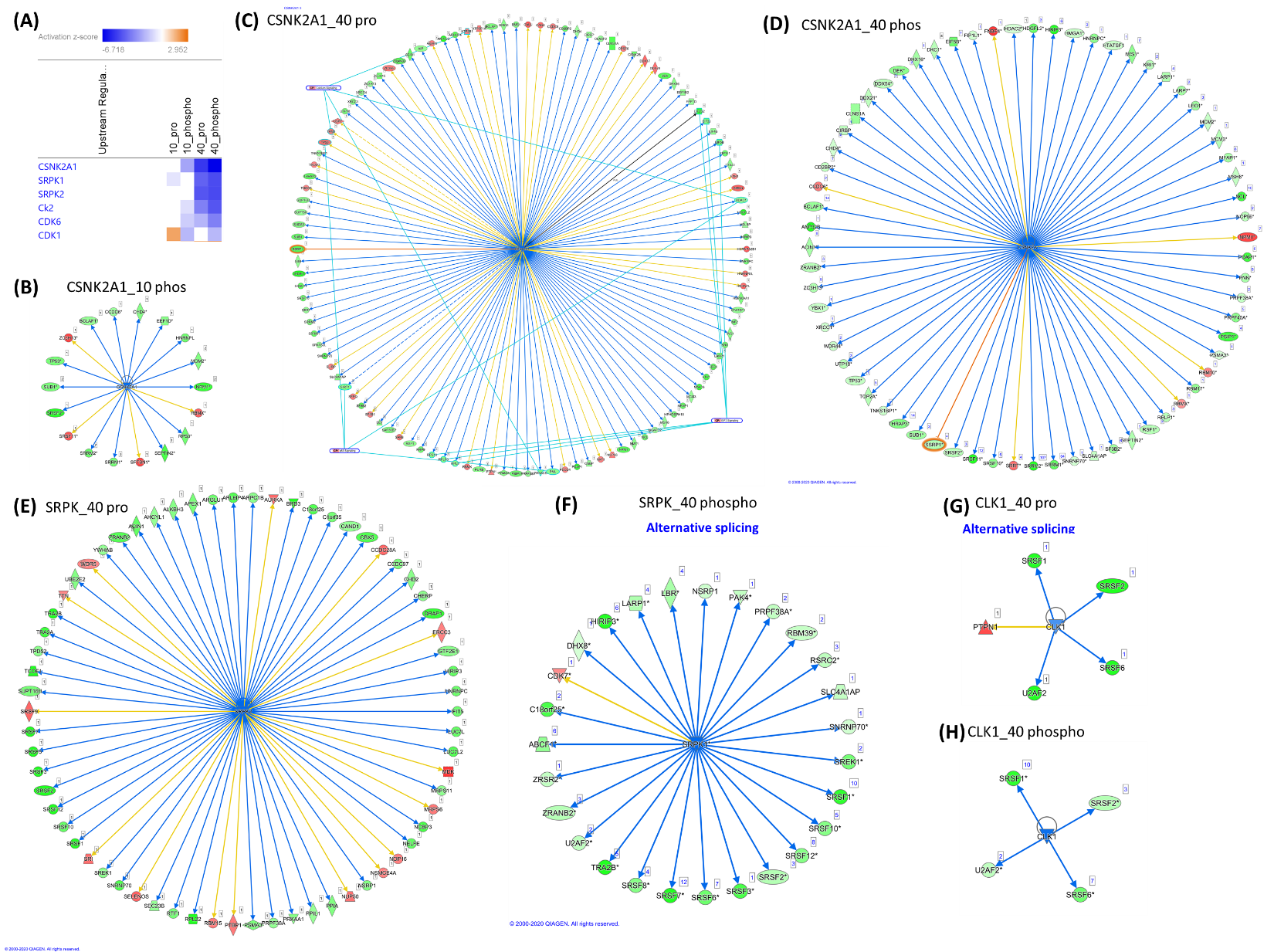
**

**Supplementary Figure 2.** Master regulators of phosphate toxicity by IPA Upstream regulator analysis. (**A**) Heatmap of upstream regulators. Orange: regulators with positive z-scores; Blue: regulators with negative z-scores; White: regulators that have z-score of 0. (**B**) CSNK2A1 regulating networks in 10mM Pi treated cells by phosphoproteomics. (**C**) CSNK2A1 regulating networks in 40mM Pi treated cells by proteomics. (**D**) CSNK2A1 regulating networks in 40mM Pi treated cells by phosphoproteomics. (**E**) SRPK regulating networks in 40mM Pi treated cells by proteomics. (**F**) SRPK regulating networks in 40mM Pi treated cells by phosphoproteomics. (**G**) CLK1 regulating networks in 40mM Pi treated cells by proteomics. (**H**) CLK1 regulating networks in 40mM Pi treated cells by phosphoproteomics. Molecules in red and green indicate up-regulated and down-regulated protein expression or phosphorylation respectively.


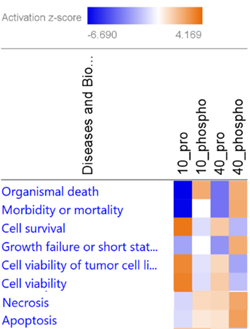


**Supplementary Figure 3.** Heatmap of excess Pi-related diseases and functions by IPA Disease and function analysis. Orange: positive z-scores indicate activation; Blue: negative z-scores indicate inactivation; White: z-score is 0, indicating that the differential gene expression data did not allow for a clear determination of the activity prediction.

**
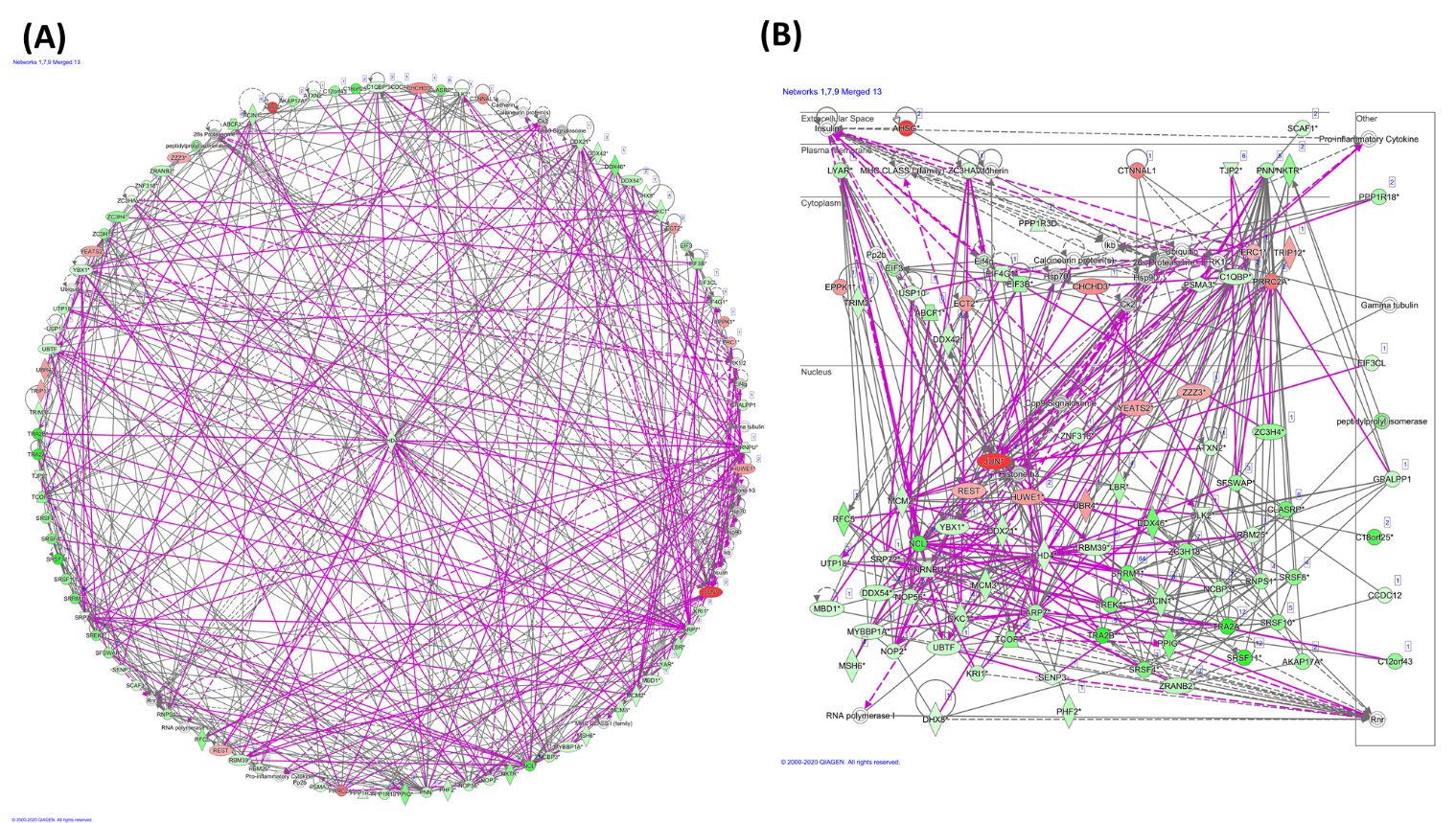
**

**Supplementary Figure 4.** Molecular networks in 40mM Pi treated cells by IPA Network analysis. (a) radial and (b) subcellular layout. Molecules in red and green indicate up-regulated and down-regulated protein expression or phosphorylation respectively.

**
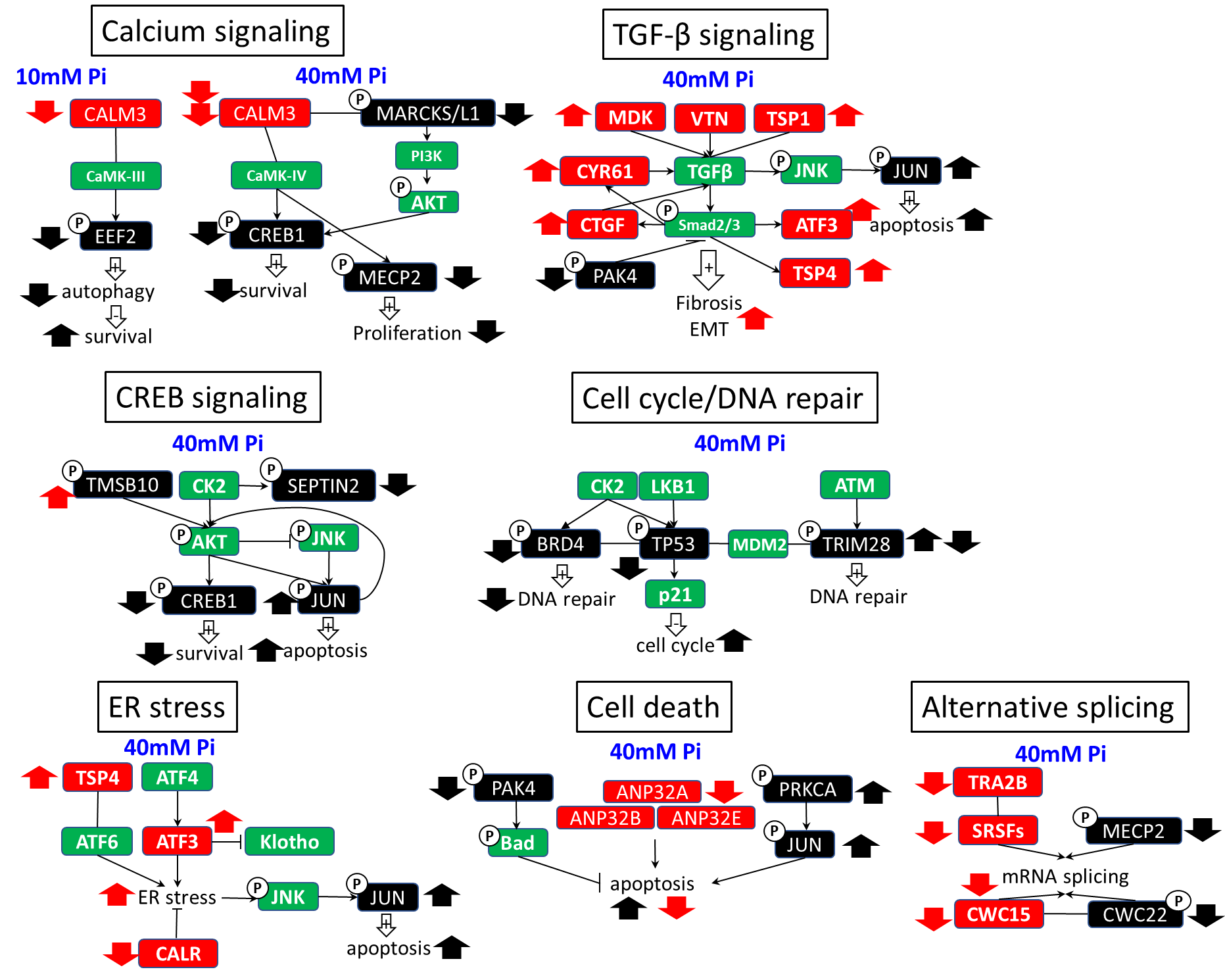
**

**Supplementary Figure 5.** High Pi-dysregulated cell signaling pathways based on literature.

**Supplementary Tables**

Table S1: Global proteomics identified proteins with at least two matching peptides.

Table S2: Protein quantitation by quantitative proteomics.

Table S3: Phosphopepite quantitation by quantitative phosphoproteomics.

Table S4: Comparison of protein fold change between 10mM and 1mM Pi treated cells.

Table S5: Comparison of protein phosphorylation fold change between 10mM and 1mM Pi treated cells.

Table S6: Comparison of protein fold change between 40mM and 1mM Pi treated cells.

Table S7: Comparison of protein phosphorylation fold change between 40mM and 1mM Pi treated cells.
